# Botulinum Neurotoxin Light Chains Expressed by Defective Herpes Simplex Virus Type-1 Vectors Cleave SNARE Proteins and Inhibit CGRP Release in Rat Sensory Neurons

**DOI:** 10.3390/toxins11020123

**Published:** 2019-02-19

**Authors:** Charles Joussain, Olivier Le Coz, Andrey Pichugin, Peggy Marconi, Filip Lim, Mariaconcetta Sicurella, Andrea Salonia, Francesco Montorsi, Francisco Wandosell, Keith Foster, François Giuliano, Alberto L. Epstein, Alejandro Aranda Muñoz

**Affiliations:** 1 UMR U1179 INSERM/Université de Versailles Saint Quentin en Yvelines (UVSQ)—Paris Saclay, 78180 Montigny-le-Bretonneux, France; charles.joussain@uvsq.fr (C.J.); olivier.le-coz@uvsq.fr (O.L.C.); karyolog@gmail.com (A.P.); francois.giuliano@uvsq.fr (F.G.); 4lejandro.4randa@gmail.com (A.A.M.); 2 Neuro-Urology R. Poincaré Hospital AP-HP, 92380 Garches, France; 3 Ipsen Innovation SAS, 91940 Les Ulis, France; 4 Department of Chemical and Pharmaceutical Sciences (DipSCF), University of Ferrara, 44121 Ferrara, Italy; peggy.marconi@unife.it (P.M.); scrmcn@unife.it (M.S.); 5 Centro de Biologia Molecular Severo Ochoa, CSIC-UAM, Universidad Autonoma de Madrid (UAM), 28049 Cantoblanco, Madrid, Spain; filip.lim@uam.es (F.L.); fwandosell@cbm.uam.es (F.W.); 6 Division of Experimental Oncology/Unit of Urology, URI, IRCCS Ospedale San Raffaele, 20129 Milan, Italy; salonia.andrea@hsr.it (A.S.); montorsi.francesco@hsr.it (F.M.); 7 University Vita-Salute San Raffaele, 20129 Milan, Italy; 8 Ipsen Bioinnovation Ltd., Abingdon, Oxon OX14 4RY, UK; keith.foster@ipsen.com

**Keywords:** HSV-1 amplicon vectors, transgenic botulinum neurotoxins, light chains, sensory neurons, DRG, SNARE proteins

## Abstract

A set of herpes simplex virus type 1 (HSV-1) amplicon vectors expressing the light chains (LC) of botulinum neurotoxins (BoNT) A, B, C, D, E and F was constructed. Their properties have been assessed in primary cultures of rat embryonic dorsal root ganglia (DRG) neurons, and in organotypic cultures of explanted DRG from adult rats. Following infection of primary cultures of rat embryonic DRG neurons, the different BoNT LC induced efficient cleavage of their corresponding target Soluble N-ethylmaleimide-sensitive-factor Attachment protein Receptor (SNARE) protein (VAMP, SNAP25, syntaxin). A similar effect was observed following infection by BoNT-A LC of organotypic cultures of adult rat DRG. To quantify and compare the functional activities of the different BoNT LC, the inhibition of calcitonin gene-related protein (CGRP) secretion was assessed in DRG neurons following infection by the different vectors. All BoNT-LC were able to inhibit CGRP secretion although to different levels. Vectors expressing BoNT-F LC displayed the highest inhibitory activity, while those expressing BoNT-D and -E LC induced a significantly lower CGRP release inhibition. Cleavage of SNARE proteins and inhibition of CGRP release could be detected in neuron cultures infected at less than one transducing unit (TU) per neuron, showing the extreme efficacy of these vectors. To our knowledge this is the first study investigating the impact of vector-expressed transgenic BoNT LC in sensory neurons.

## 1. Introduction

The clostridial neurotoxin family includes tetanus toxin (TeNT), produced by *Clostridium tetani,* and at least seven antigenically distinct botulinum neurotoxins (BoNTs) produced by different strains of *C. botulinum* (BoNT-A to -G). These proteins, which are amongst the most potent biological neurotoxins, are responsible for the conditions of tetanus and botulism, respectively. These diseases are a direct result of inhibition of calcium-dependent neurotransmitter release, a mechanism of action common to all these toxins [1]. Clostridial neurotoxins are disulphide bridge-linked proteins composed of a light chain (50 kDa), responsible for the cleavage of substrates through a zinc-dependent endoprotease activity, and a heavy chain (100 kDa) that is involved in neurospecific binding and delivery of the neurotoxin to the neuronal cytosol [2,3]. Once in the neuronal cytosol, the endopeptidase activity of the BoNTs has substrate specificity for membrane-associated proteins involved in synaptic vesicle targeting, docking, and fusion with the plasma membrane (the Soluble N-ethylmaleimide-sensitive-factor Attachment protein Receptor, or SNARE, proteins) [4]. Each neurotoxin has distinct, unique cleavage sites on their substrates. BoNT-A, BoNT-C and BoNT-E cleave synaptosomal-associated protein of 25 kDa (SNAP25), while BoNT-B, BoNT-D, BoNT-F and BoNT-G cleave vesicle-associated membrane proteins (VAMP). BoNT-C is unique in having a second substrate, syntaxin (STX) [2,5,6]. Proteolytic cleavage of any of these substrate proteins results in blockade of vesicle fusion and, in the case of the motor end plate synapse of a motoneuron, consequent inhibition of acetylcholine release [7,8,9]. The inhibition of acetylcholine release from motoneurons is responsible for the pathophysiological effects of BoNTs, leading to the impairment of striated muscle contraction. Furthermore, BoNTs are capable of inhibiting the release of neurotransmitters from a variety of synaptosomal and neuronal systems in vitro, including several neuroendocrine cells [10,11,12]. In addition to classical small molecule neurotransmitters, such as acetylcholine and noradrenaline, BoNTs also inhibit the release of neuropeptides, such as calcitonin gene-related peptide (CGRP) or substance P (SP) [13,14]. Studies using BoNTs have been carried out in a variety of cellular systems, including primary cultures of murine spinal cord neurons, bovine adreno-chromaffin cells and cerebral ganglia from the sea slug *Aplysia californica*. Cell lines such as the rat adrenal pheochromocytoma PC-12, 3T3-L1 adipocytes, insulinomas HIT- 15 and RINm5F and human neuroblastoma SH-SY5Y have also been used [12,15,16,17]. However, in spite of these studies, many aspects of the intracellular mechanism of action of BoNTs in neurons, and particularly in human sensory neurons, remain incompletely understood. Furthermore, the difficulties associated with the acquisition, storage, and manipulation of these very harmful substances, which are strictly regulated, impose considerable restrictions to their investigation.

An alternative system to the use of the whole toxins is provided by the intracellular synthesis of the BoNTs light chains (LC) using chemical or viral vectors for gene delivery, as has been demonstrated using adenovirus-based vectors expressing TeNT LC [18,19]. In the present study, nonreplicative, nontoxic amplicon vectors derived from the neurotropic herpes simplex virus type 1 (HSV-1) [20,21] were used to induce intracellular synthesis of BoNT LC, both in primary cultures of rat embryonic dorsal root ganglia (DRG) neurons and in adult rat organotypic cultures of DRG. These vectors are fully safe as the *de novo* synthesized LC cannot spread to other cells in the absence of the heavy chain [18]. Moreover, the tropism for peripheral neurons, and particularly for sensory neurons, displayed by HSV-1 [21,22] make HSV-1-derived vectors particularly well suited for investigating the properties of the transgenic LC in the appropriate cellular targets, while the reiterative nature of the amplicon vector genome [20,21] produces high-level transgene expression even at very low multiplicities of infection. Under natural in vivo conditions HSV-1 will infect mainly the oral epithelial mucosa (where the virus will accomplish its lytic, productive cycle) and then the sensory neurons innervating the infected area (where the virus will stablish latent infections) [21]. However, under experimental conditions, such as infection of cultured cells or inoculation into the stroma of specific tissues or tumors, HSV-1 vectors can transduce and express genes in other cells types as well, including central nervous system (CNS) neurons, sympathetic and parasympathetic neurons [23], and many non-neuronal tissues such as muscle cells, fibroblasts, hepatocytes, pancreatic cells, and others [21,24,25,26].

Although BoNTs are the causative agents for clinical botulism, the potent myorelaxant properties of these neurotoxins have been exploited clinically in many indications, including neurological and non-neurological disorders such as cervical dystonia [27,28], blepharospasm [29,30], spasticity [31,32], chronic migraine [33,34], idiopathic [35,36] and neurogenic detrusor overactivity [37,38], achalasia [39,40], strabismus [41,42] and pain [43,44]. Therefore, in addition to providing a useful transducing vehicle tool to study and compare the biochemical and molecular properties of BoNT LC using a fully safe and powerful system, it is likely that HSV-1 vectors expressing these toxins could be used to disrupt neurosecretion as a possible gene therapy for at least some of the above mentioned disorders, which are generally caused by aberrant or excessive neurotransmission.

We report the construction of nonreplicative HSV-1-based amplicon vectors expressing different BoNT LCs driven by a strong ubiquitous promoter, and show that these vectors express the transgenic peptides in both embryonic and adult rat sensory neurons. Each transgenic BoNT LC induces cleavage of its cognate SNARE target protein and inhibits release of neuropeptides with very high efficiency, thus perturbing neurotransmission. Our results indicate that the different BoNT LCs display differential efficacies in the inhibition of CGRP neurosecretion. 

## 2. Results

### 2.1. Structure of the Genome of Nonreplicative HSV-1 Amplicon Vectors

**Figure 1:**
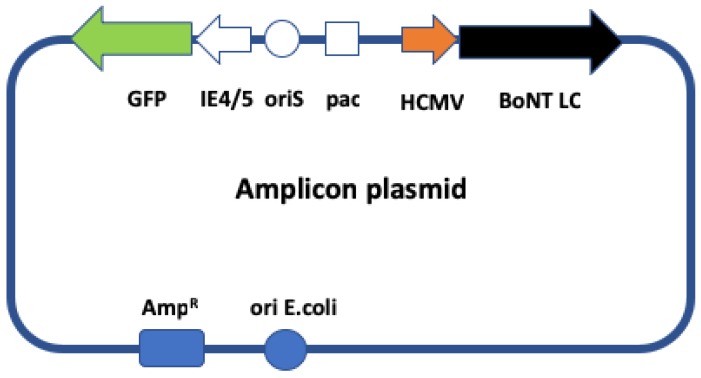
Herpes simplex virus type 1 (HSV-1)-based amplicon vectors used in this study. Scheme showing the general structure of the amplicon plasmids: oriS (white circle) and pac (white square) designate respectively the origin of viral DNA replication and the packaging signal of HSV-1. These elements allow the amplicon plasmid to be amplified and packaged in eukaryotic cells in the presence of a defective HSV-1 helper virus, thus generating the corresponding amplicon vector. Immediate early (IE)4/5 promoter (white arrow) is an HSV-1 immediate-early promoter that drives expression of the green fluorescent protein (GFP) reporter gene (green arrow), terminated by the bovine growth hormone polyadenylation signal. In addition, each amplicon plasmid expresses the light chain (LC) of one of the botulinum neurotoxins (BoNT-A to BoNT-F) or luciferase (Luc), or the GFP-BoNT-A LC fusion protein (black arrow), driven in all cases by the immediate early promoter of human cytomegalovirus (HCMV, red arrow) and terminated by the SV40 polyadenylation signal. The amplicon plasmid also contains the gene conferring ampicillin resistance (Amp^R^, blue square) and an *E. coli* DNA replication origin (blue circle) allowing the plasmid to be amplified in bacteria. The plasmid encoding the GFP-BoNT-A LC fusion protein does not encode the GFP reporter gene driven by the IE4/5 promoter.

We have constructed and produced a family of amplicon vectors expressing BoNT-A, -B, -C, -D, -E and -F LC (or luciferase as a negative control), as well as an additional vector expressing a fused GFP-BoNT-A LC. Figure 1 shows the structure of the amplicon plasmids used to generate the corresponding amplicon vectors. Each of the vectors (except for the vector encoding the fused GFP-BoNT-A LC cassette), expresses two independent transcription units. The first expresses the reporter gene GFP driven by the HSV-1 immediate-early (IE) 4/5 promoter, which is useful for measuring vector titers, to identify infected cells, and as an internal reference control for transgene expression levels. The second transcription unit expresses one of the BoNT LC (or Luc), driven in all cases by the immediate-early (IE) promoter of human cytomegalovirus (HCMV) to obtain strong and ubiquitous expression. The vector expressing the green fluorescent protein (GFP)-BoNT-A fusion protein, also driven by HCMV promoter, does not encode the IE4/5 promoter-GFP transgene cassette. Due to the reiterative character of the amplicon genome [20,21], each vector particle carries a concatemeric DNA consisting of multiple tandem repeats of the amplicon plasmid. All amplicon plasmids were sequenced to confirm the accuracy of the constructions, and all of them expressed their corresponding BoNT LC mRNA in primary cultures of rat neurons, as assessed by RT-PCR (data not shown).

### 2.2. Vectors Expressing BoNT-A LC Cleave SNAP25 in Infected Neurons

**Figure 2:**
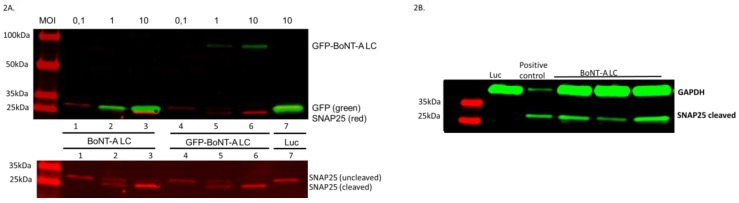
Vectors expressing BoNT-A LC cleave SNAP25 in infected rat sensory neurons. (**A**) Upper panel. Primary cultures of rat embryonic DRG neurons were infected at a multiplicity of infection (MOI) of 0.1 to 10 TU/cell, as indicated in the text, with vectors expressing BoNT-A LC (lanes 1–3), GFP-BoNT-A LC (lanes 4–6) or luciferase (Luc, lane 7). The following day, the neuronal cultures were processed for Western blotting using antibodies specific for GFP (green) or SNAP25 (red). The lower panel shows the same SNAP25 fragments slightly overexposed in red and without the green GFP labels to facilitate detection of the SNAP25 fragments. (**B**) Adult rat DRG organotypic cultures which had undergone neurite outgrowth (usually three days after explant) were infected with 2 × 10^6^ TU of vectors expressing BoNT-A LC or luciferase (Luc). Three days later, culture samples were processed for Western blots using an antibody that specifically recognizes the cleaved form of the SNAP25 protein. As a loading control, an antibody specific for the cellular protein glyceraldehyde 3-phosphate dehydrogenase (GAPDH) was used. Extracts from three different ganglia are shown. The positive control lane corresponds to a sample of cultured rat embryonic sensory neurons that have been infected at a MOI of 10 TU/cell with the same vector and probed using the same antibody.

The first set of experiments was designed to assess whether the amplicon vector BoNT-A LC was able to express this protein in sensory neurons and whether the transgenic protein did induce cleavage of its cognate SNAP25 target protein. Since there are no available commercial antibodies recognizing any of the BoNT LC, we first used transcription cassettes in which the BoNTs LC were labelled by a His tag. However, we were unable to detect any peptide using anti-His specific antibodies, although this same antibody was able to recognize a His-tagged tetanus neurotoxin (TeNT, data not shown). Therefore, we constructed a vector expressing a GFP N-terminal fusion to the BoNT-A LC (GFP-BoNT-A LC) to assess the presence of the fused protein in infected neurons. To this end, and also to assess whether the fused and the non-fused BoNT-A LC proteins were able to cleave SNAP25, primary cultures of rat embryonic DRG neurons were infected by vectors GFP-BoNT-A LC, BoNT-A LC, or Luc (the two latter vectors also expressing GFP as a free protein) at increasing multiplicities of infection (MOI), from 0.1 to 10.0 transducing units (TU)/neuron. At 24 h post-infection (pi) the neuronal cultures were processed for Western blotting using antibodies specific for GFP and antibodies that recognize both the native (about 25 kDa) and the cleaved (about 24 kDa) forms of SNAP25. In Figure 2A, GFP is shown in green and SNAP25 is shown in red as both proteins have very similar molecular weights. The upper panel of Figure 2A shows a dose-dependent expression of proteins that are recognized by GFP-specific antibodies and which correspond either to the GFP-BoNT-A LC fused protein (about 75 kDa, lanes 4–6) or to the free GFP (about 25 kDa, lanes 1–3 and 7) confirming dose-dependent transgene expression in the infected DRG neurons. Importantly, this figure also shows that infection with GFP-BoNT-A LC and BoNT-A LC vectors induced a dose-dependent cleavage of SNAP25, demonstrating that both proteins are functional. Thus, at MOI of 0.1 TU/cell mostly the native form of SNAP25 is observed (lanes 1 and 4), while at a MOI of 10 TU/cell only the cleaved form can be observed (lanes 3 and 6). At intermediate MOI (1 TU/cell) both the native and cleaved forms of SNAP25 can be observed (lanes 2 and 5). The negative control expressing luciferase induced no cleavage of SNAP25 at the highest MOI (lane 7). The lower panel of this figure shows the red SNAP25 bands independently of the green bands, to facilitate detection of the SNAP25 fragments.

Having demonstrated that cleavage of SNAP25 was a *bona fide* surrogate for the expression of BoNT-A in infected cells, we performed a further experiment in which organotypic cultures of adult rat DRG were infected with 2 × 10^6^ TU per explanted ganglion using vectors expressing BoNT-A LC or Luc. Three days later, the explanted DRG cultures (three independent experiments are shown in Figure 2B), were processed for Western blotting using antibodies that specifically recognize only the cleaved form of SNAP25 (kindly donated by IPSEN Bioinnovation Ltd.). We used this antibody in these tests because in the case of organotypic DRG cultures most cells would escape infection (only the outgrowing neurites could be infected) and the presence of large amounts of noncleaved SNAP25 from noninfected cells could mask the results. As a positive control for this antibody we used rat embryonic sensory neuron cultures infected with the BoNT-A LC vector. Figure 2B shows that a band corresponding to the cleaved form of SNAP25 is visible in the organotypic culture of adult DRG, thus confirming the functionality of BoNT-A LC, not only in cultured embryonic DRG neurons but also in explanted DRG from adult animals. Altogether, these experiments clearly showed expression and functionality of BoNT-A LC encoded by the amplicon vector, both in embryonic and adult rat DRG neurons, thereby indicating that BoNT-A LC was able to cleave its cognate SNARE target protein SNAP25 when intracellularly synthesized in HSV-1 amplicon vector-infected neurons.

### 2.3. Vectors Expressing BoNT-A LC Inhibit Secretion of CGRP Neuropeptide in Infected Neurons

**Figure 3:**
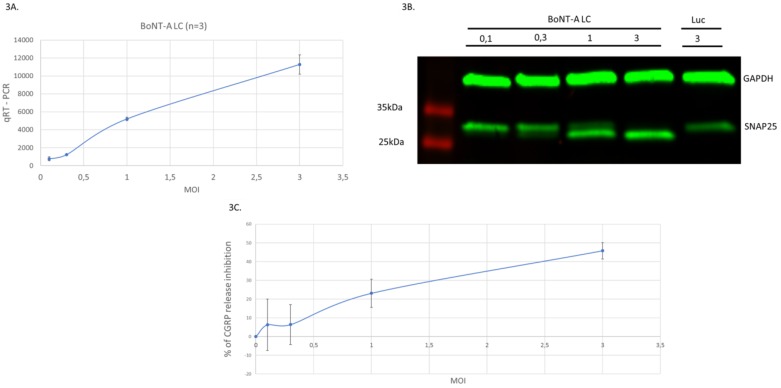
Vectors expressing BoNT-A LC inhibit CGRP release in infected neurons. Cultures of rat embryonic sensory neurons were infected with vector expressing BoNT-A LC at different MOIs as indicated in the text. The following day, culture samples were processed for qRT-PCR, Western blot and ELISA. (**A**) qRT-PCR of RNA from cell extracts showing a vector dose-dependent increase of BoNT-A LC expression. Primers used are described in Material and Methods. Means and standard deviation were calculated for each MOI. (**B**) Western blots of cell extracts, using antibodies specific for the cellular protein GAPDH or for SNAP25, showing the vector dose-dependent increase in SNAP25 cleavage. (**C**) Inhibition of CGRP release expressed as the inverse of the percentage of inhibition of CGRP release induced by the BoNT-A LC vector with respect to that induced by the vector expressing Luc. A vector dose-dependent increase in CGRP release inhibition can be observed. Means and standard deviation were calculated for each MOI.

Following cleavage of one or more of the SNARE proteins by BoNTs, synaptic vesicles cannot anymore fuse with presynaptic membranes, thereby resulting in inhibition of neurosecretion and neurotransmission. To investigate whether expression of BoNT-A LC from HSV-1 vectors in cultured rat embryonic sensory neurons results in inhibition of neurosecretion, we assessed amounts of CGRP in extracellular medium following vector infection. Since basal levels of CGRP secretion by sensory neurons are low, the impact of vector infection was investigated in neurons previously treated with KCl, which is known to induce CGRP release [45]. Primary cultures of rat embryonic sensory neurons were infected at increasing MOI (from 0.1 to 3.0 TU/cell) with vectors expressing BoNT-A LC or Luc. The following day, three different tests were performed: from a first group of infected neurons (a pool of three wells of infected cells/MOI), RNA was extracted and qRT-PCR was performed to assess levels of BoNT-A LC RNA expression. As expected, there is a dose-dependent increase in RNA expression following infection at increasing MOIs (Figure 3A). A second group of neurons (also a pool of 3 wells/MOI) was used to perform Western blotting (Figure 3B) using the antibodies that recognize both the native and cleaved forms of SNAP25, yielding similar results to those shown in Figure 2A, with significant cleavage of SNAP25 starting at a very low MOI (from 0.3 TU/cell). Lastly, in these same neuronal samples, before RNA extraction and Western blotting, 75 mM KCl was first added to the culture media and samples of the media were taken 30 min later to assess the extracellular levels of CGRP using specific ELISA (Figure 3C). Results shown in Figure 3C indicate that the vector expressing BoNT-A LC was able to induce a dose-dependent inhibition of CGRP secretion. In contrast, in neurons infected with vectors expressing luciferase, or in control noninfected neurons, no inhibition of CGRP release took place (data not shown). Significant inhibition of CGRP release can be observed starting at a MOI of 1 TU/cell. Therefore, these results show a dose-dependent increase in RNA expression, in SNAP25 cleavage, and in inhibition of CGRP release, clearly indicating that transgenic expression of BoNT-A LC from HSV-1 amplicon vectors in cultured sensory neurons elicited both cleavage of SNAP25 and inhibition of CGRP release.

### 2.4. All BoNT LC Types Display Cleavage of Their Cognate SNARE Protein and Inhibition of CGRP Release

**Figure 4:**
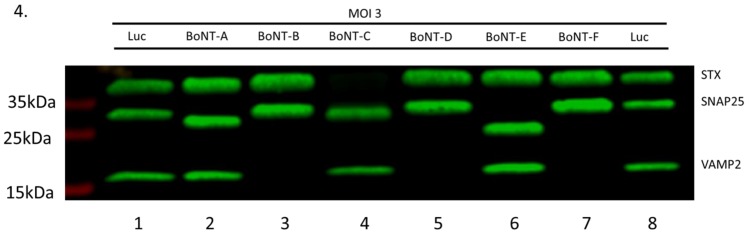
Each transgenic BoNT LC cleaves its cognate SNARE protein. Primary cultures of rat sensory DRG neurons were infected at MOI of 3 TU/cell with vectors expressing the LC of BoNT-A, -B, -C, -D, -E, -F or Luc, as control. The following day culture samples were processed for Western blot using antibodies specific for each of the three SNARE proteins (SNAP25, VAMP2 and STX). It can be observed in this figure that the LC of BoNT-A, -C, and -E cleave SNAP25 and that the cleaved products in each case display a slightly different migration, corresponding to different SNAP25 cleavage sites targeted by each of these three neurotoxins. Vectors expressing the LC of BoNT-B, -D and -F result in cleavage of VAMP2, rendering this protein undetectable by the VAMP2-specific antibody. Similarly, STX is also undetectable in extracts from neuronal cultures infected by the vector expressing BoNT-C. In the lanes labelled Luc the positions on the blot of native STX, SNAP25 and VAMP2 can be observed.

The next set of experiments was designed to extend the above results to other BoNT LC and to comparatively assess their expression and activities in infected sensory neurons. Firstly, cultures of embryonic rat sensory neurons were infected with vectors expressing the LC of BoNT-A, -B, -C, -D, -E, -F, or Luc, at a MOI of 3 TU/cell. Western blots of cell extracts performed the next day show that the cognate SNARE proteins targeted by the different BoNT LC were cleaved in all cases (Figure 4). Therefore, SNAP25 was cleaved by vectors expressing the LC of BoNT-A, -C and -E (lanes 2, 4 and 6), while VAMP2 was cleaved by vectors expressing the LC of BoNT-B, -D and -F (lanes 3, 5 and 7). As expected, BoNT-C LC also cleaved syntaxin (STX) in addition to SNAP25 (lane 4), being the only BoNT able to cleave two different SNARE proteins [5,6]. BoNT-A, -C and -E cleave SNAP25 at different places, explaining the small differences in size of the cleaved products as compared to noncleaved SNAP25. Since the cleavage fragments of VAMP2 and STX are rapidly degraded following cleavage [46], these proteins are no longer visible in the Western blot.

**Figure 5:**
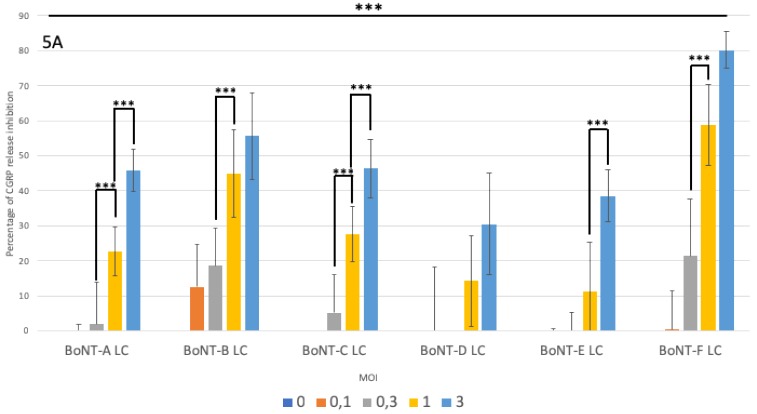

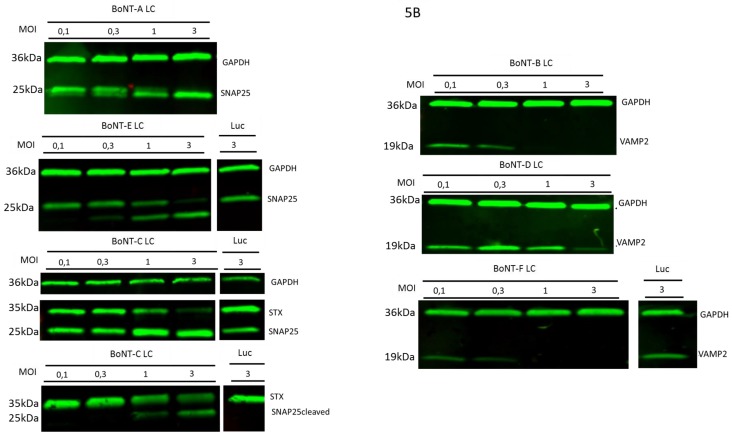
All transgenic BoNT LC types inhibit CGRP release from infected neurons. Primary cultures of rat sensory neurons were infected at increasing MOI, from 0.1 to 3.0 TU/cell, with vectors expressing the different toxins. The following day, culture samples were processed for ELISA and Western blots. (**A**) Inhibition of CGRP release expressed as the inverse of the percentage of inhibition of CGRP release induced by each BoNT LC vector with respect to that induced by the vector expressing Luc. For each vector, a dose-dependent increase in CGRP release inhibition can be observed, although the potency of inhibition by each vector varies. Statistical analysis was performed using the Student’s *t*-test, *** corresponding to *p* < 0.001 (GraphPad Prism v5). (**B**) Western blots of cell extracts using antibodies specific for the cellular protein GAPDH or for STX, VAMP2 and the native and cleaved forms of SNAP25. For each vector, a dose-dependent increase in the level of SNARE protein cleavage can be observed.

In a second experiment, cultured rat embryonic DRG neurons were infected with vectors expressing the LC of BoNT-A, -B, -C, -D, -E, -F, or Luc at increasing MOI (from 0.3 to 3.0 TU/cell). On the following day, microphotographs of GFP autofluorescence were taken to confirm that all cultures had been infected at comparable levels (data not shown). As in the experiment described in Figure 3, cell samples were treated with KCl to induce CGRP secretion. Then neurons were harvested for Western blots to assess cleavage of SNARE proteins. Results shown in Figure 5A indicate that all BoNT LC were able to inhibit CGRP release in a vector dose-dependent manner, although they did so with variable potencies. Thus, the vector expressing BoNT-F LC produced a greater inhibition of CGRP release than those expressing BoNT-B or -D LC, which also cleave VAMP2. Indeed, at a MOI of 1 TU/cell BoNT-F LC inhibits CGRP release as efficiently as BoNT-B LC at 3 TU/cell and is more potent than BoNT-D LC at 3 TU/cell. Regarding the BoNTs that cleave SNAP25, no significative differences were evident between BoNT-A, BoNT-C and BoNT-E LCs in inhibiting CGRP secretion. It must be noted however that BoNT-C LC cleaves STX in addition to SNAP25, and this could also contribute to inhibition of neurosecretion [5,6]. In mock-infected embryonic rat sensory neurons or in neurons infected with a vector expressing Luc, no inhibition of CGRP release took place (data not shown). It is noteworthy that inhibition of CGRP release can be detected in some cases in neurons infected at MOI lower than 1 TU/cell, suggesting that a single infecting vector particle per cell was able to synthesize enough neurotoxin for neurosecretion inhibition, thus highlighting the potency of these vectors as a source of intracellular toxin.

By analyzing cleavage of the SNARE proteins (Figure 5B) at different MOI, it was found in all experiments that levels of the native SNAP25, VAMP2, and STX proteins progressively decrease with the infectious dose and the native forms of these proteins are no longer visible in the gel at the highest MOI. We then investigated whether a relationship could be established between potency of inhibition of CGRP release and levels of cleavage of the SNARE proteins. As shown in Figure 5B, considering cleavage of SNAP25, the blot shows that BoNT-A LC cleaved significant amounts of SNAP25 even at 0.3 TU/cell and at 3 TU/cell native SNAP25 is no more detectable, while with BoNT-E LC native SNAP25 was still detectable even at 3 TU/cell. The effect of BoNT-C LC is harder to analyze due to the small difference in molecular weight between the native and cleaved forms of SNAP25. Accordingly, with BoNT-C LC expressing vectors we have used the antibody recognizing only the cleaved form. It came out that detectable amounts of cleaved SNAP25 were observed at 1 TU/cell. Cleavage of STX was also found at 1.0 TU/cell with BoNT-C LC expressing vector. Regarding the BoNTs that cleave VAMP2, BoNT-B and -F LC displayed a similar behavior, with VAMP2 becoming undetectable at MOI as low as 1 TU/cell, while cleavage of VAMP2 by vector expressing BoNT-D LC is much lower, becoming clearly evident only at a MOI of 3 TU/cell. Taken together, these results indicate that the level of inhibition of CGRP release and cleavage of SNARE proteins both increase with the dose of the infecting vector, and suggest a reasonable correlation between both parameters. It is noteworthy from Figure 5A,B that a significant proportion of CGRP is still released even under conditions where SNARE proteins are no longer detectable. In these experiments, BoNT-F LC reproducibly appeared as the most potent inhibitor of CGRP release when expressed from HSV-1-based amplicon vectors in embryonic rat sensory neurons.

## 3. Discussion

Nonreplicative, nontoxic amplicon vectors derived from neurotropic HSV-1, expressing the LC of botulinum neurotoxins, BoNT-A, -B, -C, -D, -E and -F, all of them driven by the strong HCMV IE promoter, have been constructed and assessed. Results reported in this study indicate that these vectors were able to infect embryonic and adult rat DRG neurons, respectively as primary cultures and as organotypic cultures, and that the transgenic BoNT LCs were expressed in all cases, thereby cleaving their cognate SNARE protein and significantly decreasing neurosecretion of CGRP in embryonic rat sensory neurons. Boulis and coworkers previously described the use of adenovirus vectors to study properties of the light chains of the related tetanus neurotoxin [18,19]. As far as we know however, our study is the first to investigate and compare the effects of transgenic BoNT LC, following infection from neurotropic HSV-1-based vectors, or from any other viral vector. 

Native BoNT-A holotoxin, delivered as an extracellular protein, is currently being used both for clinical and cosmetic applications and there is much interest in further understanding its molecular effects in eukaryotic neurons and in comparing its biological properties to those of other BoNT serotypes. Our approach, consisting in directly expressing transgenic BoNT LC in neurons, could be very useful in this regard as it allows the study of effects of the harmless light chain in the absence of the heavy chain, particularly as the native protein is extremely toxic and cannot be used by research laboratories without specific authorization. In addition, variability between the different BoNTs could be due at least in part to differences at the level of toxin binding and penetration, which relies on the properties of the heavy chain. It should be noted however that exogenously administrated holotoxins and vector-induced synthesis of transgenic LC could differ in several quantitative and qualitative ways, including expression, concentration, localization, trafficking, solubility, and ubiquitin-mediated degradation [47]. Indeed these differences could explain some discrepancies between our results and those obtained using holotoxins [47]. Botulinum neurotoxins, both when secreted by bacteria through natural infection or injected for clinical or cosmetic indications, attach to the synaptic membrane of neurons and are internalized into endosomes. From there the protein is cleaved and the light chain is released into the synaptic region, close to the SNARE exocytotic apparatus, where the toxins will exert their endopeptidase activities, cleaving their cognate targets and disrupting neurosecretion and neurotransmission [1,2,3,4]. In the case of the vector-induced transgenic expression used in our approach, and although we have not performed in depth molecular studies, it can be assumed that the transgenes are transcribed in the nucleus, and that the mRNA will be translated in the soma of the transduced neurons. Since BoNTs are bacterial proteins, having no eukaryotic localization signals, they should most likely disseminate homogenously within the whole neuron cytoplasm, without displaying any preferential localization to synaptic regions. Yet, the transgenic BoNT LC were able to efficiently cleave their target proteins and to inhibit neurotransmitter release, suggesting that a significant fraction of them were either able to reach the synaptic region to interact with their target or, alternatively, that the toxins could bind and cleave their target proteins before they reach the synaptic region. More studies using specific antibodies for the toxins are needed in order to address this point. If the BoNTs actually bind the SNARE proteins only in the synaptic region, it should be possible to increase the fraction of transduced neurotoxin localized to this region by introducing synaptic-region localizing signals to the toxins, thereby improving their trafficking to the synaptic region and increasing their efficacy. This is currently being done in our laboratory. These vectors could be very promising tools to study BoNT-LC trafficking and physiology in neuronal cells.

The present data show clear significant biological effects induced by transgenic BoNT LCs synthesized in neurons, as both cleavage of the corresponding SNARE proteins and inhibition of neuropeptide release could be observed even at very low multiplicities of infection. Other studies have already demonstrated a correlation between cleavage of SNARE proteins and inhibition of neurotransmitter release following administration of BoNT holotoxins [48]. Remarkably, in our case, cultured neurons infected at less than 1 TU/cell exhibit a significant inhibition of CGRP release, pointing out the efficacy of these vectors. This likely results from the ability of HSV-1 to efficiently penetrate and be retrogradely transported in sensory neurons, combined with the intrinsic properties of amplicon vectors, which produces very high expression levels due to the reiterative structure of the vector genome [20,21,22]. However, results presented in Figure 5 also show that some level of CGRP release persisted even in conditions where the SNARE proteins are no longer detectable. Although we have not directly addressed this point, it is likely that the residual release of CGRP could be explained by the presence of undetectable amounts of native, non-cleaved SNARE proteins.

Our approach could be an efficient alternative to the supply of the native toxins in specific future therapeutics approaches. Indeed, BoNT-A and BoNT-B are already approved for a variety of indications, while other applications are presently in development [49]. However, direct administration of BoNTs, although having represented a very important breakthrough in the treatment of some pathologies, still displays important drawbacks and limitations, such as the need to regularly repeat the treatment, since the half-life of the protein is limited, or the fact that in some cases, some resistance could appear [50]. Our strategy could therefore become a preferred approach to induce continued intracellular synthesis of the BoNT-LC, thereby resolving at least some of these limitations. Although in the present study BoNT expression was driven by the transient HCMV promoter, thereby not allowing long-term studies, the very large genomic capacity of HSV-1 vectors permits the introduction of viral or cellular genetic elements able to confer long-term transgenic expression, as well as neuron-specific promoters, or even inducible promoters, to drive transgene expression, thus allowing stable, selective, or controllable, BoNT LC expression in different types of neurons. This could open new perspectives both in the understanding of the basic biology of BoNT-LCs, as well as for therapeutic development.

## 4. Materials and Methods

### 4.1. Primary Cultures of Embryonic Rat Dorsal Root Ganglia (DRG) Neurons 

Pregnant female adult Sprague-Dawley rats (250 g, E14–E18) were sacrificed in accordance with the European Communities Council Directives 2010/63/UE on the use of laboratory animal and care regulation in force in France (Ministry of Agriculture, Authorization Agreement No. A78-322-3, 18 December 2013 and B78-423-1, 28 July 2017). Embryonic rat DRG were dissected in a Petri dish containing sterile ice-cold Hanks balanced salt solution containing 0.4% dextrose (HBSS-D). Sensory neurons were dissociated in trypsin/EDTA solution and incubated 20–40 min at 37 °C, before being seeded in poly-D-Lysine (0.1 mg/mL) coated microplates (Nunc 96 wells) at a density of 50.000 cell per well in 100 µL of Neurobasal Sup Medium (Neurobasal medium, 5–10% inactivated fetal bovine serum (iFBS), 2% B27, 2 mM L-glutamine, 50 U/mL streptomycin and penicillin, and 250 ng/mL NGF). After 45 min 200 µL of Neurobasal Sup Medium was added. Cells were cultured in an incubator (5% CO_2_ at 37 °C). On the third day of culture, culture media were removed and replaced by 200 µL of Neurobasal Sup Medium with the addition of the mitotic inhibitor cytosine arabinoside (10 µM) until the fourth day. After 4 days of culture, media were removed and the vectors, diluted in 200 µL of fresh culture medium, were added at the MOI indicated in the text. 

### 4.2. Organotypic Cultures of Rat Adult DRG

Female adult rats (250 g) (Sprague-Dawley) were sacrificed in accordance with the European Communities Council Directives 2010/63/UE on the use of laboratory animal and care regulation in force in France (Ministry of Agriculture, Authorization Agreement No. A78-322-3, 18 December 2013 and B78-423-1, 28 July 2017). After death, a dorsal middle incision was performed to expose S3-L4 vertebrae. Supraspinous, interspinous, and yellow ligaments, were sectioned. A laminectomy was then performed to expose the spinal cord and L4 to S2 DRG. S1 and L6 DRG were harvested separately and immediately put into a M24 plate precoated with 15 to 20 µL of Matrigel (SIGMA, Saint Louis, MO, USA) 30 min before plating ganglia. Neurobasal Medium supplemented with 5–10% iFBS was then added to overlay the ganglia. Media were renewed every 2 days. After 3 days, once neurite outgrowth allowed infection of internal neurons, a suspension of 2 × 10^6^ vector particles was added directly to the ganglia and left until the end of experimentation.

### 4.3. Construction and Titration of Nonreplicative HSV-1 Amplicon Vectors

#### 4.3.1. Plasmids Containing the LC of BoNTs 

A family of pUC57 plasmids carrying the sequences of the light chains of BoNT-A, -B, -C, -D, -E, and –F, as described in GeneBank [accession numbers of BoNT-A LC: DL193752.1; BoNT-B LC: KF878257.1; BoNT-C LC: X53751.1; BoNT-D LC: AB012112; BoNT-E LC: EF028403.1; BoNT-F LC: GU213203.1] has been ordered (GenScript). An additional pUC57 plasmid, carrying the sequence of GFP-BoNT-A LC fused protein was also constructed. Since the BoNT genes are of bacterial origin, the sequences carried by the pUC57 plasmids were modified to take into account the codon usage of mammalian cells, thus optimizing translation in these cells but without modifying the translated product. In addition all these sequences were engineered to contain a NheI site at the 5′ end and a NotI site at the 3′ end to facilitate subcloning into amplicon plasmids.

#### 4.3.2. Generation of Amplicon Plasmids 

A set of HSV-1 amplicon plasmids carrying the sequences of the BoNT LC, or firefly luciferase as control, driven in all cases by the immediate-early promoter of human cytomegalovirus (HCMV) enhancer/promoter, was constructed. For this, the pUC57 plasmids were digested by NheI/NotI and the fragments carrying the neurotoxin coding regions were cloned into the NheI/NotI sites of amplicon plasmid pA-EUA1 [51] thus generating the amplicon plasmids BoNT-A, -B, -C, -D, -E, and –F respectively, as illustrated in Figure 1. It is noteworthy that these amplicon plasmids also express a GFP reporter gene driven by the IE4/5 immediate-early promoter of HSV-1 [51]. In addition an amplicon plasmid was constructed to express the GFP-BoNT-A LC fusion protein. This plasmid was constructed by sub-cloning the GFP-BoNT-A LC fragment into the NheI/NotI sites of pA-SK, an amplicon plasmid very similar to pA-EUA1, except that it does not contain the GFP transcription cassette [52].

#### 4.3.3. Production and Titration of Amplicon Vector Particles 

Amplicon vector stocks were prepared as already described, using the highly neuro-attenuated, nonreplicative HSV-1LaLΔJ virus as helper [52]. Briefly, 7B cells [53] were independently transfected with 5 µg of each amplicon plasmid using Lipofectamine Plus (Thermofisher, Invitrogen, Carlsbad, CA, USA). One day later, transfected cells were superinfected at a MOI of 0.3 plaque forming unit (PFU) per cell, with HSV-1LaLΔJ helper virus. When the cytopathic effect (CPE) was maximal, cells were collected by centrifugation, disrupted by three freeze/thaw cycles to release vector stocks, and re-centrifuged at 1000× *g* for 10 min to pellet the cell debris. Helper and vector particles in the supernatants were then titrated as already described [49]. Cells expressing fluorescent GFP were scored at 24 h postinfection (pi) using an inverted fluorescence microscope (Olympus, Tokyo, Japan) yielding transducing units (TU). Titers of helper virus particles are provided as plaque-forming units (PFU) per millilitre, as already described [49]. Serial passages of the vector populations were then carried out on 7B cells, thus increasing the TU/PFU ratio to 99/1 or higher.

#### 4.3.4. Infection Procedure 

After 4 days of culture of primary culture of rat embryonic DRG neurons, or once explanted DRG displayed neurite outgrowth (usually after 3 days in culture), the culture medium was withdrawn and replaced with a minimal amount of medium containing the vector suspension. After two hours of contact, fresh medium was added to the wells and the cultures were kept at 37 °C in a 5% CO_2_ atmosphere until the end of the experiments.

#### 4.3.5. qRT-PCR

At 24hpi culture media were removed and cells were resuspended into RLT buffer containing 10% β-mercaptoethanol. RNA was then extracted using Qiagen RNeasy MiniKit (Qiagen GmbH, Hilden, Germany) and cDNA was obtained using SuperscriptIII (Thermofisher, Invitrogen, Carlsbad, CA, USA), qRT-PCR was performed using GoTaq G2 Colorless Mix and using the oligonucleotides shown in Table 1 (Eurogentech, Liege, Belgium). Results were normalized with respect to the cellular house-keeping gene GAPDH.

#### 4.3.6. Western Blots (WB) 

At 24hpi, culture medium was removed and cells were resuspended into RIPA lysis buffer (Thermofisher, Rockford, IL, USA). After cell lysis, homogenates were centrifuged at 10,000 rpm for 10 min at 4 °C. Cell lysates were resuspended into Laemmli buffer (Bio-Rad Laboratories Inc, Hercules, CA, USA). After protein quantification, 50 µg of protein was used to perform electrophoresis (Tris-HCl PAGE-Gel, Bio-Rad laboratories Inc, Hercules, CA, USA). Transfer of proteins was performed using activated-methanol membrane (Immobilon FL Transfer Membrane, Merck Millipore, Tullagreen, Carrigtwohill, County Cork, Ireland) at 4 °C during 1h30 with 0.35A. Blocking and antibody incubation (shown in Table 2) were performed using the IBind^TM^ Flex Western System SFL2000 (Thermofisher, Invitrogen, Carlsbad, CA, USA). The intensity of each band was determined by pixel density integration using LI-COR^®^ Odyssey Western Blotting Kits.

#### 4.3.7. ELISA

Primary cultures of embryonic rat DRG neurons were infected at increasing MOI, as indicated in the text, with amplicon vectors expressing the different BoNT LC. At 24hpi, cultured neurons were stimulated with 75 mM KCl to induce CGRP release [45]. Thirty minutes before and thirty minutes following KCl treatment, 100 microliter aliquots were taken from the culture media and assessed for the presence of CGRP by ELISA (using the CGRP ELISA kit from Spi Bio, ref N° A05482, Montigny-le-Bretonneux, France, Statistical analysis was performed using the Student t test, *** corresponding to *p* < 0.001 (GraphPad Prism v5, San Diego, CA, USA).

### 4.4. Note on Biosafety and Biosecurity

There is currently significant concern about the potential misuse of vectors expressing toxins or toxic proteins in general [54]. Accordingly, all genetic constructs used in this study were declared as genetic modified organisms (GMO) expressing the LC of BoNTs to the French regulatory agency (authorization file N° 3910) and the study was conducted in accordance to the safety and confinement requirements established by the French regulatory authorities.

Although, as quoted in Introduction, Clostridium neurotoxins are amongst the most potent biological neurotoxins, the use of HSV-1-derived vectors expressing BoNT LC under the control of HCMV promoter appears to be safe for several reasons. (i) Many studies have demonstrated that the use of isolated LC does not pose a threat since these proteolytic fragments do not cross cellular membranes and are not capable of neuronal binding and uptake without the HC [18,19]. (ii) The amplicon vectors used in this study are fully defective HSV-1-derived vectors, encoding no viral lytic genes and inducing no destruction of the infected cells. These vectors are unable to replicate in the absence of a helper virus that provides structural and replicative functions [20,21,22]. The helper virus used in this study is itself defective [52] and can replicate only in cells, such as the 7B cells used in this study, which complement in trans the essential functions that have been deleted from the helper virus genome [53]. This prevents multiplication and spread of the vectors outside the complementing cells. (iii) The HCMV promoter that drives expression of the BoNTs LC is well known to be a strong and ubiquitous but transient promoter when expressed from HSV-1 gene transfer platform [55], meaning that expression is completely silenced between 2 or 3 weeks postinfection, both in in vitro and in vivo systems.

## Figures and Tables

**Table 1:** Oligonucleotides used in the qRT-PCR reactions.

Name of Oligonucleotide	Sequence of Oligonucleotide
RT-BoNT-A F1	GCGCCGACATCATCCAGTTC
RT-BoNT-A R1	GTGTCCACCTCCAGGCTCTC
Rat.GAPDH-1049F	GTGGACCTCATGGCCTACAT
Rat.GAPDH-1190R	TGTGAGGGAGATGCTCAGTG

**Table 2:** Primary and secondary antibodies used in the Western blots.

**Primary Antibodies**	**Brand**	**Reference**	**Species**
STX	ABCAM	ab3265	Rabbit
SNAP25	ABCAM	ab66066	Rabbit
VAMP2	ABCAM	ab3347	Rabbit
SNAP25	IPSEN		Rabbit
GAPDH	Millipore	CB1001	Mouse
GFP	Life technologies	A6455	Rabbit
**Secondary Antibodies**	**Brand**	**Reference**	**Species**
Mouse (800)	Li-Cor	926-32210	Goat
Rabbit (680)	Li-Cor	926-68071	Goat
Rabbit (800)	Li-Cor	926-32211	Goat
